# Association between catheter ablation and psychiatric disorder risk in adults with atrial fibrillation: a multi-institutional retrospective cohort study

**DOI:** 10.3389/fpsyt.2025.1467876

**Published:** 2025-03-21

**Authors:** Ting-Hui Liu, Jheng-Yan Wu, Po-Yu Huang, Wan-Hsuan Hsu, Min-Hsiang Chuang, Ya-Wen Tsai, Kuang-Yang Hsieh, Chih-Cheng Lai

**Affiliations:** ^1^ Department of Psychiatry, Chi Mei Medical Center, Tainan, Taiwan; ^2^ Department of Nutrition, Chi Mei Medical Center, Tainan, Taiwan; ^3^ Department of Internal Medicine, Chi Mei Medical Center, Tainan, Taiwan; ^4^ Center for Integrative Medicine, Chi Mei Medical Center, Tainan, Taiwan; ^5^ Division of Hospital Medicine, Department of Internal Medicine, Chi Mei Medical Center, Tainan, Taiwan; ^6^ School of Medicine, College of Medicine, National Sun Yat-sen University, Kaohsiung, Taiwan

**Keywords:** atrial fibrillation, catheter ablation, anxiety, depression, insomnia, suicidal ideation or attempt, dementia

## Abstract

**Background:**

Given that atrial fibrillation (AF) s associated with a high risk of psychiatric disorders, understanding the potential benefits of catheter ablation is clinically significant. This study was conducted to examine whether catheter ablation can prevent psychiatric disorders in patients with AF.

**Methods:**

A retrospective cohort study was conducted over two years using data from the TriNetX electronic health record network. The study included adults diagnosed with AF and treated with either antiarrhythmic or rate-control medications. Participants were divided into two groups: those who underwent catheter ablation and a control group without ablation. The primary outcome measured was a composite of anxiety, depression, and insomnia occurrence within one to three years post-treatment. Secondary outcomes included individual psychiatric disorders, suicidal ideation or attempts, dementia, cerebral infarction, and atopic dermatitis (as a negative control).

**Results:**

We included 21,019 patients in each matched group. The ablation group demonstrated a lower risk of the primary combined outcome (hazard ratio(HR):0.873, 95% confidence interval (CI) 0.784–0.973, p<0.01), and secondary outcomes including anxiety (HR:0.822, 95% CI:0.700–0.964; p=0.016), depression (HR:0.614, 95% CI:0.508–0.743; p<0.001), suicidal ideation or attempts (HR:0.392, 95% CI:0.165–0.934; p=0.028), dementia (HR:0.569, 95% CI:0.422–0.767; p<0.001), and cerebral infarction (HR:0.704, 95% CI:0.622–0.797; p<0.001) compared to the non-ablation group.

**Conclusions:**

In patients with atrial fibrillation, catheter ablation was associated with a reduced risk of developing psychiatric disorders, including anxiety, depression, insomnia, suicidal ideation or attempt, and dementia, in comparison to those who did not undergo ablation. Clinicians should consider incorporating psychiatric risk factors into their comprehensive patient assessment when evaluating candidates for catheter ablation.

## Introduction

Atrial fibrillation (AF) is a prevalent form of symptomatic arrhythmia, impacting approximately 59.7 million individuals worldwide (1). This condition not only predisposes affected individuals to severe cardiovascular complications but also substantially diminishes their overall quality of life (2, 3). While the connection between AF and cardiovascular complications like stroke and heart failure is well-documented (4, 5), growing research reveals its significant impact on mental health (6–9). Patients with AF show higher rates of anxiety, depression, and other psychiatric disorders – a link that may be explained by several biological mechanisms. These include chronic inflammation, disrupted blood flow patterns, and the physical and emotional stress of managing a chronic heart condition (7, 10, 11). Understanding these connections is crucial, as mental health challenges can significantly affect how patients manage their AF, follow treatment plans, and perceive their illness, ultimately influencing their overall health outcomes.

Catheter ablation, a therapeutic modality that aims to restore sinus rhythm by isolating the pulmonary veins, has demonstrated promising results in reducing AF burden and associated complications (12). Randomized controlled trials (RCTs) have provided evidence that catheter ablation is a well-tolerated procedure for managing AF in patients who exhibit non-responsiveness to antiarrhythmic drugs (13, 14). Moreover, recent evidence from RCTs suggests that, in comparison to antiarrhythmic drugs, using catheter ablation as an initial treatment can help reduce the recurrence of AF and the need for hospitalizations (15, 16).

Beyond its antiarrhythmic benefits, there is a growing interest in exploring whether catheter ablation can potentially reduce the risk of psychiatric disorders in adults with AF. A recent multicenter RCT known as the Randomized Evaluation of the Impact of Catheter Ablation on Psychological Distress in Atrial Fibrillation (REMEDIAL) trial revealed that catheter ablation for AF led to a significant improvement in anxiety, depression, and psychological distress when compared to medication management (17). It is worth noting that this trial had a relatively small cohort size, including only 100 individuals. At present, there is a noticeable absence of large-scale real-world data that investigate the impact of catheter ablation on psychiatric disorders in patients with AF.

To address this research gap, we conducted a retrospective cohort study using TriNetX, a global health research network that provides comprehensive, updated, and longitudinal patient data from healthcare organizations. Our study aimed to assess the association between catheter ablation and psychiatric disorder risk in adults with AF.

## Methods

### Data source

This retrospective cohort study used data from the TriNetX research network, providing access to the de-identified records of 250 million unique patients sourced from over 120 healthcare organizations (HCOs) spanning 19 countries. TriNetX represents a global coalition of health research entities that gather anonymized patient data from electronic health records (EHRs). The dataset includes an extensive array of patient information such as demographics, clinical diagnoses, performed procedures, prescription details, laboratory findings, genetic information, and documentation of healthcare organization visits. In this analysis, we used Research Network that includes data on more than 30 million patients from 80 healthcare organizations. TriNetX facilitates patient-level data analysis through integrated tools and provides researchers with synthesized results (18, 19). Access to comprehensive information in the database is available online. The requirement for written informed consent was exempted due to the anonymized nature of the TriNetX data. The study protocol received approval from the Institutional Review Board of Chi Mei Medical Center (approval no. 11206-E02).

### Patient selection

The TriNetX Research database comprising 80 HCOs as of October 15, 2023, was used in the patient selection process. We began with individuals who visited HCOs more than twice. The inclusion criteria for our cohort were an age of at least 18 years, a diagnosis of AF, and a documented history of receiving antiarrhythmic or rate-control medications. AF was defined according to the International Classification of Diseases, Tenth Revision, Clinical Modification (ICD-10-CM) code I48.0 (Paroxysmal atrial fibrillation), I48.1 (Persistent atrial fibrillation), and I48.2 (Chronic atrial fibrillation) (20). We employed RxNorm, ATC, and VA codes to ascertain the usage of antiarrhythmic or rate-control medications. Next, the cohort was categorized into two groups based on the treatment type: those who underwent catheter ablation and those who did not. Catheter ablation was identified using the procedure codes listed in **Supplementary Tables S1**, **S2**. Patients with psychiatric diagnoses one year before and one year after the index date were excluded. The index date, as defined for each cohort (further details are available in **Supplementary Tables S3**, **S4**), signifies the date when patients first fulfilled the specific cohort criteria. For the ablation group, this date corresponds to the procedure date, while for the non-ablation group, it corresponds to the date of diagnosis. Further details regarding the inclusion and exclusion criteria, and additional patient information, are provided in **Supplementary Tables S1 and S2**.

Finally, the patient selection involved the use of propensity score matching on a 1:1 basis, matching for age at the index, race, sex, and comorbid medical conditions. This resulted in two comparable groups for the study: the ablation and non-ablation groups.

### Covariates

To mitigate baseline characteristic disparities between the ablation and non-ablation cohorts, we accounted for 23 variables (21, 22). The covariates employed for 1:1 propensity score matching encompassed age at index event, gender (female or male), race (Caucasian, African American, Asian, or indeterminate), along with a range of comorbid conditions. These conditions comprised hypertension, diabetes mellitus, hyperlipidemia, chronic kidney disease, ischemic heart disease, heart failure, cerebral infarction, atherosclerosis, peripheral vascular diseases, neoplasms, chronic obstructive pulmonary disease (COPD), asthma, chronic liver diseases, substance use disorders, and alcohol-related disorders. This comprehensive approach aimed to ensure a rigorous comparison and enhance the reliability of the study outcomes.

### Outcome measurement

The primary outcome of our study was the composite incidence of specific psychiatric disorders, including anxiety, depression, and insomnia within a one- to three-year period following the index date. The index date, defined for each cohort (details provided in **Supplementary Tables S3**, **S4**), marked the day when patients initially met the specified cohort criteria (23). These particular psychiatric disorders, including anxiety, depression, and insomnia, were identified according on ICD-10-CM as previously described (**Supplementary Table S5**) (24).

For secondary outcomes, we examined each of these psychiatric disorders individually, along with outcomes related to suicidal ideation or attempts, and dementia. Additionally, we included two outcome categories to provide a comprehensive assessment. The first, serving as a positive control, was cerebral infarction, while the second, serving as a negative control, was atopic dermatitis (25). This approach allowed us to thoroughly evaluate the impact of interventions across a diverse range of health outcomes.

### Statistical analysis

The TriNetX platform’s integrated propensity score-matching function was employed to establish a 1:1 correspondence between subjects in both the ablation and non-ablation groups. The matching was executed using a nearest-neighbor greedy algorithm with a caliper limit set at 0.1 of the pooled standard deviation, optimizing the pairing of participants. Subsequently, standardized differences were calculated to evaluate the equivalence of the matched groups, with absolute values below 0.1 denoting a robust match (26).

Subsequently, we performed a Kaplan-Meier analysis, followed by log-rank tests with calculations of hazard ratios (HR) at 95% confidence intervals (CI) to compare the two groups (27). Statistical significance was set at p< 0.05. HR was used to describe the relative risk of outcomes by comparing time-to-event rates and was calculated using a proportional hazard model, which is a built-in function in TriNetX (28).

For the subgroup analysis, we compared the primary and secondary outcomes between the two groups, stratified by age (18–64 and ≥65 years), sex (male and female), race (white and non-white), and presence of comorbidities (paroxysmal AF, non-paroxysmal AF, heart failure, cancer, COPD, diabetes mellitus, dyslipidemia, and chronic liver disease) (20, 29).

## Results

### Baseline characteristics

**Figure 1:**
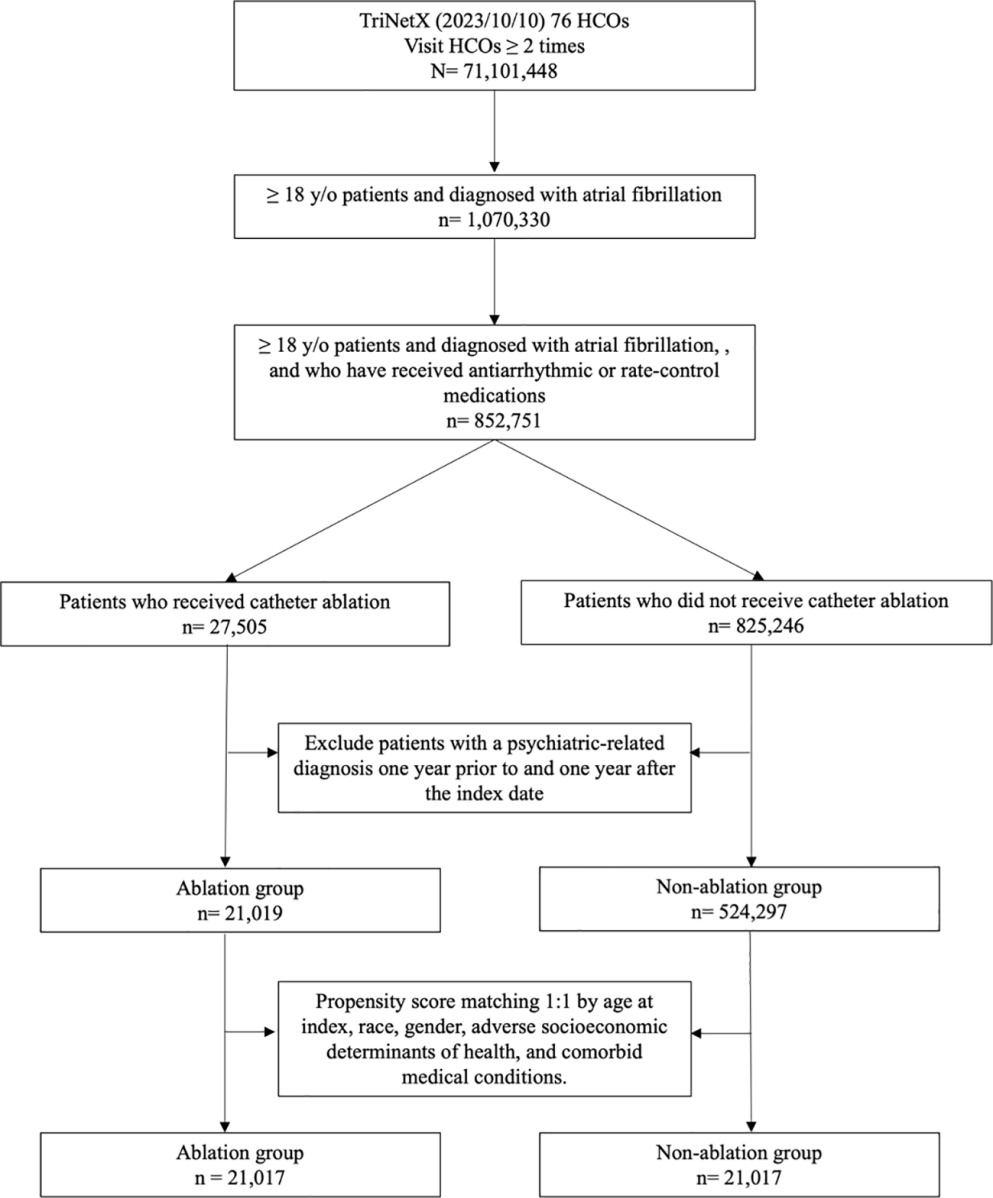
Flowchart of patient selection and cohort construction of the cohort.

The final cohort comprised 21,019 individuals who underwent catheter ablation and 21,017 individuals who did not undergo ablation, serving as the comparator cohort (**Figure 1**). The baseline characteristics differed significantly between the ablation and non-ablation groups. The mean age at index for the ablation group was 63.9 ± 10.4 years, whereas the non-ablation group exhibited an older demographic with a mean age of 71.5 ± 12.0 years. The sex distribution in both groups before matching showed a higher proportion of males (68.6% and 59.5% in the ablation and non-ablation groups, respectively). In terms of racial demographics, the majority were Caucasian, constituting 86.64% of the ablation group and 77.69% of the non-ablation group. Comorbidity analysis revealed that hypertensive diseases were the most prevalent, affecting 50.35% and 43.35% of the patients in the ablation and non-ablation groups, respectively. Dyslipidemia was also present, with rates of 37.47 and 33.13% in the ablation and non-ablation groups, respectively. Regarding comorbidities, conditions such as peripheral vascular diseases, neoplasms, and COPD were more common in the non-ablation group. The matching process substantially minimized the demographic and comorbidity disparities between the two groups, achieving balance through propensity score matching. (**Table 1**).

**Table 1:** Comparison of characteristics of ablation group and non-ablation group before and after matching.

	Before matching			After matching		
	Ablation group (n= 21,019)	Non-ablation group (n= 524,297)	Std diff.	Ablation group (n= 21,017)	Non-ablation group (n= 21,017)	Std diff.
**Age at Index**	63.9 ± 10.4	71.5 ± 12.0	0.676	63.9 ± 10.4	64.1 ± 11.0	0.019
Sex						
Male	14495 (68.96)	312052 (59.59)	0.197	14493 (68.96)	14480 (68.9)	0.001
Female	6172 (29.36)	204596 (39.07)	0.206	6172 (29.37)	6200 (29.5)	0.003
Race						
White	18211 (86.64)	412071 (78.69)	0.211	18209 (86.64)	18330 (87.22)	0.017
Black or African American	724 (3.45)	39094 (7.47)	0.178	724 (3.45)	691 (3.29)	0.009
Asian	312 (1.48)	14970 (2.86)	0.094	312 (1.49)	295 (1.4)	0.007
Native Hawaiian or Other Pacific Islander	35 (0.17)	2781 (0.53)	0.062	35 (0.17)	35 (0.17)	0.000
Unknown Race	1721 (8.19)	53926 (10.3)	0.073	1721 (8.19)	1654 (7.87)	0.012
Comorbidities						
Hypertensive diseases	10582 (50.35)	228283 (43.59)	0.136	10580 (50.34)	10660 (50.72)	0.008
Diabetes mellitus	2804 (13.34)	89356 (17.06)	0.104	2804 (13.34)	2828 (13.46)	0.003
Hyperlipidemia	7876 (37.47)	173483 (33.13)	0.091	7875 (37.47)	7832 (37.27)	0.004
Chronic kidney disease	1261 (6)	58111 (11.1)	0.183	1261 (6)	1254 (5.97)	0.001
Ischemic heart diseases	5204 (24.76)	120714 (23.05)	0.040	5203 (24.76)	5195 (24.72)	0.001
Heart failure	3640 (17.32)	85657 (16.36)	0.026	3639 (17.32)	3489 (16.6)	0.019
Cerebral infarction	527 (2.51)	20430 (3.9)	0.079	527 (2.51)	475 (2.26)	0.016
Atherosclerosis	927 (4.41)	23517 (4.49)	0.004	927 (4.41)	824 (3.92)	0.025
Peripheral vascular diseases	524 (2.49)	24766 (4.73)	0.120	524 (2.49)	467 (2.22)	0.018
Neoplasms	2634 (12.53)	91479 (17.47)	0.139	2634 (12.53)	2548 (12.12)	0.012
Chronic obstructive pulmonary disease	917 (4.36)	38144 (7.28)	0.125	917 (4.36)	856 (4.07)	0.014
Asthma	1025 (4.88)	20469 (3.91)	0.047	1025 (4.88)	900 (4.28)	0.028
Chronic liver disease	660 (3.14)	19106 (3.65)	0.028	660 (3.14)	542 (2.58)	0.034
Substance use disorders	1416 (6.74)	30234 (5.77)	0.040	1416 (6.74)	1265 (6.02)	0.029
Alcohol related disorders	392 (1.87)	7400 (1.41)	0.036	392 (1.87)	336 (1.6)	0.020

### Primary outcomes

**Figure 2:**
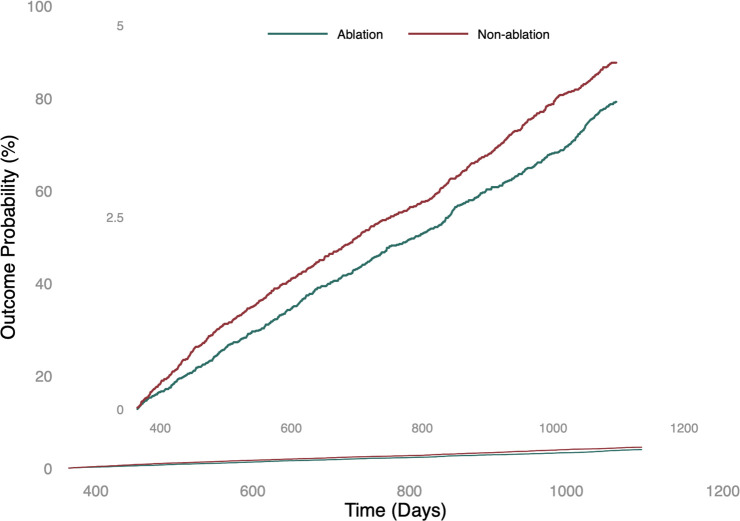
The probability of the primary outcome: a composite of anxiety, depression, and insomnia.

In our propensity score-matched cohorts, the ablation group exhibited a reduced incidence of psychiatric disorders compared to the non-ablation group, with an HR of 0.873 (95% CI, 0.784–0.973) (**Table 2**). Specifically, during the follow-up period, 598 individuals (2.8%) with AF who underwent catheter ablation developed various psychiatric disorders, including anxiety, depression, and insomnia. In contrast, 728 individuals representing 3.5% of the group with AF who did not undergo catheter ablation had these disorders. These differences persisted consistently throughout the follow-up period, indicating that the ablation group had a lower risk of developing psychiatric disorders than the non-ablation group (log-rank p < 0.01; **Figure 2**).

**Table 2:** The hazard ratio and cases number for comparing matched ablation group and non-ablation group for the primary composite outcome and secondary outcomes.

Outcomes	Case number		Hazard ratio	(95%CI)	P value
	Ablation group (n= 21,017)	Non-ablation group (n= 21,017)			
**Any psychiatric disorders**	598	728	0.873	(0.784, 0.973)	**0.014**
Anxiety	264	345	0.822	(0.700, 0.964)	**0.016**
Depression	168	293	0.614	(0.508, 0.743)	**<.001**
Insomnia	398	470	0.896	(0.784, 1.024)	0.108
Suicide ideation or attempt	10	19	0.392	(0.165, 0.934)	**0.028**
Dementia	66	124	0.569	(0.422, 0.767)	**<.001**
Negative control- Atopic dermatitis	41	35	1.248	(0.795, 1.959)	0.335
Positive control- Cerebral infarction	420	626	0.704	(0.622, 0.797)	**<.001**

Bold values indicate the primary outcome of our study, which was the composite incidence of specific psychiatric disorders, including anxiety, depression, and insomnia. Additionally, bold values denote results that reached statistical significance.

### Secondary outcomes

Throughout the follow-up period, the ablation group had 264 cases of anxiety, whereas the non-ablation group had 345 cases, resulting in an HR of 0.822 (95% CI:0.700–0.964; p=0.016). In the context of depression, there were 168 cases in the ablation group and 293 cases in the non-ablation group, with an HR of 0.614 (95% CI:0.508–0.743; p<0.001). Regarding suicidal ideation or attempts, there were 10 cases in the ablation group versus 19 cases in the non-ablation group, and the HR was 0.392 (95% CI:0.165–0.934; p=0.028). Regarding dementia, there were 66 cases in the ablation group and 124 cases in the non-ablation group, yielding an HR of 0.569 (95% CI:0.422–0.767; p<0.001). The HR for atopic dermatitis, the negative control, was 1.248 (95% CI:0.795–1.959; p=0.335). Eventually, for the positive control (cerebral infarction), the HR was 0.704 (95% CI:0.622–0.797; p<0.001).

### Subgroup analyses

**Figure 3:**
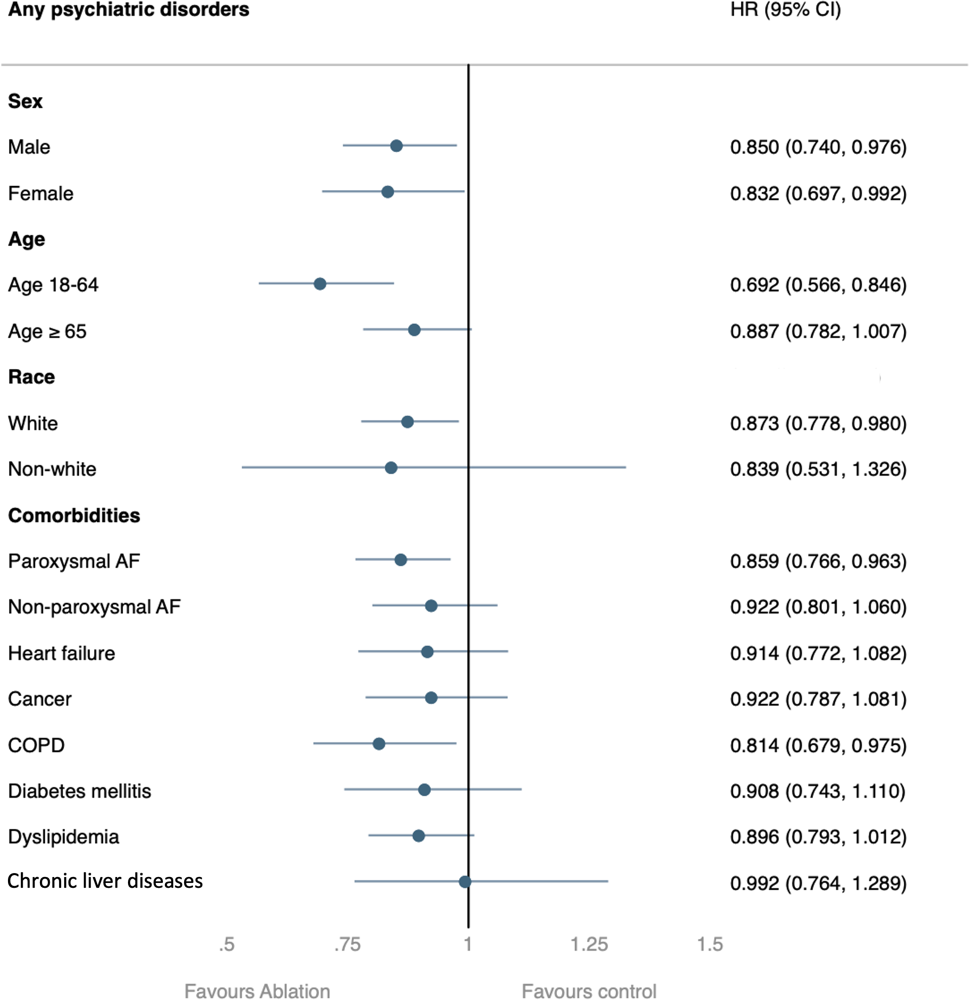
Subgroup analysis of the risk of the primary outcome between the ablation group and non-ablation group. AF: atrial fibrillation; COPD: chronic obstruction pulmonary disease.

In most subgroup analyses, the ablation group showed a consistently and significantly lower risk of developing psychiatric disorders (**Figure 3**). However, there were certain exceptions, including those aged > 65 years, non-Caucasian populations, and those with certain comorbidities. In these cases, only a non-significant lower risk of psychiatric disorders was observed.

In terms of individual outcomes, the analysis revealed that the ablation group exhibited a significantly lower risk of anxiety than the non-ablation group, particularly in the 18–64 years age subgroup and among patients with paroxysmal AF (**Supplementary Figure S1**). Concerning depression, most subgroups demonstrated a significantly lower risk in the ablation group, except for non-Caucasian individuals and those with liver diseases (**Supplementary Figure S2**). In the case of insomnia, only the 18–64 years age subgroup, patients with paroxysmal atrial fibrillation, and those with COPD demonstrated a reduced risk in the ablation group (**Supplementary Figure S3**). However, no significant differences were observed in suicide risk across the subgroups (**Supplementary Figure S4**). In the case of dementia, the majority of subgroups exhibited a significantly lower risk in the ablation group, except for the 18–64 age group, non-Caucasian individuals, patients with diabetes mellitus, and those with chronic liver diseases (**Supplementary Figure S5**). In the context of the negative control, atopic dermatitis, no significant differences were found between groups in any of the subgroup analyses (**Supplementary Figure S6**). Finally, for the positive control outcome of cerebral infarction, all subgroups showed a significantly lower risk in the ablation group than in the non-ablation group, except for patients with chronic liver diseases (**Supplementary Figure S7**).

## Discussion

This is the first large-scale retrospective cohort study involving 42,034 individuals to assess the effects of catheter ablation on psychiatric disorders in patients with AF. Our primary finding was that patients who underwent catheter ablation after AF diagnosis demonstrated a lower risk of psychiatric disorders. These patients were less likely to experience depression, anxiety, or insomnia than those who did not undergo catheter ablation. Importantly, this protective effect was consistently observed across different demographic subgroups, including sex, race (Caucasian or non-Caucasian), age group between 18–59 years, as well as patients with paroxysmal AF or COPD. These findings suggest that catheter ablation may serve as a protective measure to reduce the risk of psychiatric disorders in patients with AF.

Notably, our results align with those of the REMEDIAL trial conducted by Al Kaisey et al. In their study involving participants with symptomatic AF, improvements in psychological symptoms such as anxiety and depression were observed following catheter ablation, but not with medical therapy (17). Although our study shares a common conclusion with the REMEDIAL trial, it is important to acknowledge the methodological differences between the two. Notably, our study had a larger cohort size and employed diagnostic codes as opposed to rating scales to measure the outcomes. Moreover, our present work, portrays the real-world scenario, offering insights that effectively reflect the everyday clinical practice, capturing a broader range of patient populations with uncontrolled variables, thus complementing the controlled environment of randomized trials. Despite their methodological disparities, both studies conducted using varying approaches have consistently demonstrated the potential benefits of catheter ablation in preventing psychiatric disorders in patients with AF, thereby reinforcing the robustness of this conclusion.

Our secondary findings indicate that catheter ablation significantly reduces the risk of various psychiatric conditions, such as anxiety, depression, insomnia, suicidal ideation or attempt, and dementia, in patients with AF compared to those who did not undergo ablation. For a comprehensive understanding of the potential mechanisms underlying this improvement, a study conducted by Hasebe et al. discovered that augmentation of parasympathetic reactivity to stress was correlated with reduced anxiety (30). This implies that modifications in cardiac autonomic function may play a pivotal role in the psychological improvement experienced by patients after undergoing catheter ablation for AF. Additionally, it is possible that by restoring regular heart rhythm, catheter ablation leads to improved cerebral blood flow- thereby potentially alleviating symptoms of depression, anxiety, and cognitive disorders (31, 32). Furthermore, the consequent decrease in systemic inflammation, commonly associated with AF, might further play a role in reducing psychiatric symptoms (32). Studies have demonstrated that systemic inflammation might be a shared mechanism contributing to psychiatric manifestations (33). Together, these findings suggest that catheter ablation’s efficacy in mitigating psychiatric conditions might stem from its ability to modulate cardiac autonomic function, enhance cerebral blood flow through rhythm regularization, and reduce systemic inflammation (34).

The observed variations in psychiatric risk reduction following catheter ablation across different subgroups suggest that its benefits on insomnia and dementia risk are influenced by neurophysiological, autonomic, and inflammatory mechanisms, as well as patient-specific disease burden (35–38). Our findings indicate that younger adults (18–64 years), those with paroxysmal AF, and patients with COPD experienced significant reductions in insomnia risk, likely due to greater neuroplasticity, enhanced autonomic regulation, and improved cerebrovascular reserve, which facilitate recovery from AF-related hypoperfusion, oxidative stress, and systemic inflammation (37, 39–42). In COPD, the elimination of AF-related sympathetic overactivation and intermittent hypoxia may stabilize sleep and cerebral oxygenation, reducing neuropsychiatric complications (43, 44). However, dementia risk reduction was more evident in older adults (≥65 years), white patients, and those with cardiovascular comorbidities. This may reflect the greater cumulative impact of AF-related hypoperfusion and microembolic events in these populations, where sinus rhythm restoration prevents further neurovascular injury (45, 46).

In contrast, younger patients, those with DM, and individuals with liver disease did not show significant dementia risk reduction, suggesting that the protective effects of ablation may be counteracted by pre-existing metabolic and systemic conditions. Younger individuals may have a lower baseline dementia risk and greater neuroplasticity, making short-term cognitive benefits less detectable (46, 47). However, in DM and liver disease, persistent systemic inflammation, insulin resistance, endothelial dysfunction, and neurotoxic metabolic effects may continue to drive cognitive decline regardless of AF status (48–51). Given that diabetes is a major risk factor for both vascular and Alzheimer’s-type dementia, the metabolic and microvascular changes associated with DM may override the cognitive benefits of sinus rhythm restoration (52, 53). Similarly, liver disease is associated with hepatic encephalopathy, altered ammonia metabolism, and systemic inflammation, all of which can contribute to dementia independent of AF (51, 54). These findings highlight the complex interplay between cardiovascular function, metabolic disease, and brain health, highlighting the need for prospective studies to investigate the mechanisms linking catheter ablation to psychiatric outcomes across diverse patient populations.

Our study revealed that patients who underwent catheter ablation exhibited a lower risk of experiencing suicidal ideation or attempting suicide than those who did not undergo catheter ablation. This finding aligns with that of a previous cohort study conducted by Walters et al., which involved 78 patients and revealed a high prevalence of severe psychological distress (35%) and suicidal ideation (20%) among individuals with AF (9). Notably, they found that effective catheter ablation of AF was associated with a substantial improvement in both psychological distress and reported suicidal ideation. However, a key distinction between our study and that of Walters et al. lies in our cohort selection. We excluded individuals with a prior history of psychiatric disorders and focused on new-onset psychiatric conditions following a diagnosis of AF. This design choice ensured that our findings were pertinent to the development of psychiatric disorders after an AF diagnosis, offering valuable insights into this specific patient condition.

### Strengths and limitations

This is the first large-scale retrospective cohort study to evaluate the preventive effect of catheter ablation on psychiatric disorders in patients with AF. Despite potential biases, such as misdiagnosis or documentation errors associated with registry databases, we leveraged a comprehensive and dynamically updated population-based database. This enabled us to examine diverse global populations within the current timeframe. While propensity score matching helps balance observed covariates, we acknowledge that unobserved confounders, such as socioeconomic status, medication adherence, may still exist. To minimize this residual confounding, we conducted several sensitivity analyses and included multiple relevant covariates in our matching process. Additionally, the possibility of selective reporting of results must be considered; however, we minimized this risk by reporting all measured outcomes, including both significant and non-significant findings, to ensure transparency. Our methodological approach was further strengthened by the use of both positive and negative controls, with the latter helping validate our analytical methods. For instance, the non-significant finding for atopic dermatitis (p=0.335) serves as an appropriate negative control, as catheter ablation would not be expected to affect dermatological conditions. We specifically excluded patients with prior psychiatric disorders to ensure clean ascertainment of new-onset conditions, which strengthens our ability to establish temporal relationships, though we acknowledge this may limit generalizability to the broader AF population. While our use of diagnostic codes follows established methodology in large-scale epidemiological studies, we recognize this limits the precision of psychiatric assessment. All HR for individual psychiatric outcomes were statistically significant and less than 1, with the exception of insomnia. For insomnia, although the hazard ratio was less than one (suggesting a protective effect of catheter ablation), the result was not statistically significant (p=0.108), possibly due to limited statistical power. Similarly, despite finding a significantly reduced risk of insomnia in the subgroups of young adults, patients with COPD, and paroxysmal AF, non-significant findings were observed in subgroups with other comorbidities and older adults. This may be attributed to the smaller sample sizes in these subgroups. Further large-scale studies are warranted to assess the association with various psychiatric diseases among specific subgroups with a small sample size in this study.

## Conclusions

This retrospective cohort study demonstrated that catheter ablation in patients with AF might be associated with a significantly lower risk of developing psychiatric disorders, including anxiety, depression, insomnia, suicidal ideation or attempt, and dementia, compared to those who did not undergo ablation. While these findings suggest potential psychiatric benefits beyond the established cardiovascular advantages of ablation, further prospective studies are needed to confirm whether this association should influence the timing of ablation decisions. When evaluating candidates for catheter ablation, clinicians may consider incorporating psychiatric risk factors as part of their comprehensive patient assessment, alongside established clinical indicators.

## Data Availability

The original contributions presented in the study are included in the article/**Supplementary Material**. Further inquiries can be directed to the corresponding authors.
