# Supplementary material for: Association between catheter ablation and psychiatric disorder risk in adults with atrial fibrillation: a multi-institutional retrospective cohort study
Source: Front Psychiatry. 2025 Mar 21;16:1467876. doi: 10.3389/fpsyt.2025.1467876 (PMC11969046; doi:10.3389/fpsyt.2025.1467876)

**Supplementary methods**

# Introduction

TriNetX is the global federated health research network providing access to electronic medical records (diagnoses, procedures, medications, laboratory values, genomic information) across large healthcare organizations (HCOs). This report was run on the set of HCOs grouped into a network called COVID-19 Research Network. This network included 88 HCO(s).

# Methods

The analysis process includes two main steps: 1) Defining the cohorts through query criteria; 2) Setting up and running the analysis. Setting up the analysis requires definitions for the index event, outcomes criteria, and the time frame. Compare outcomes supports four analyses: Measures of Association, Survival, Number of Instances and Lab result distribution. These analyses have additional options that are listed in the Outcomes Definitions and Analyses Specifications section below. Furthermore, characteristics of the cohorts that are balanced using propensity score matching are also included in the Propensity Score Matching section.

## Cohorts definition

This section lists all terms used in the definitions of the two cohorts.

**Table S1:** Query Criteria for Cohort (query name: ablation group)

|  | | | | | |
| --- | --- | --- | --- | --- | --- |
| Ungrouped terms | | | | | |
|  | must have |  | demographics | Age | Age (at least 18 years (most recent occurrence)) |
| Group 1 | | | | | |
|  | **visit** | | | | |
|  | must have |  | visit | TNX:Visit | Visit |
|  | number of occurrences | | Greater than or equal to 2 instances | | |
|  | date constraint | | The terms in this group occurred at any time | | |
| Group 2 | | | | | |
|  | **Group 2A Atrial Fibrillation** | | | | |
|  | must have | any of | diagnosis | UMLS:ICD10CM:I48.0 | Paroxysmal atrial fibrillation |
|  |  |  | diagnosis | UMLS:ICD10CM:I48.1 | Persistent atrial fibrillation |
|  |  |  | diagnosis | UMLS:ICD10CM:I48.2 | Chronic atrial fibrillation |
|  | date constraint | | This group occurred before 3 years ago | | |
|  | event relationship | | Any instance of medication occurred on or after the first instance of Atrial Fibrillation | | |
|  | **Group 2B medication** | | | | |
|  | must have | any of | medication | NLM:ATC:C01B | ANTIARRHYTHMICS, CLASS I AND III |
|  |  |  | medication | NLM:VA:CV300 | ANTIARRHYTHMICS |
|  |  |  | medication | NLM:ATC:C07 | BETA BLOCKING AGENTS |
|  |  |  | medication | NLM:VA:CV100 | BETA BLOCKERS/RELATED |
|  |  |  | medication | NLM:VA:CV100 | BETA BLOCKERS/RELATED |
|  |  |  | medication | NLM:VA:CV200 | CALCIUM CHANNEL BLOCKERS |
|  |  |  | medication | NLM:ATC:C08 | CALCIUM CHANNEL BLOCKERS |
|  |  |  | medication | NLM:ATC:C01A | CARDIAC GLYCOSIDES |
|  |  |  | medication | NLM:RXNORM:4441 | flecainide |
|  |  |  | medication | NLM:RXNORM:8754 | propafenone |
|  |  |  | medication | NLM:RXNORM:4441 | flecainide |
|  |  |  | medication | NLM:RXNORM:9947 | sotalol |
|  |  |  | medication | NLM:RXNORM:703 | amiodarone |
|  |  |  | medication | NLM:RXNORM:233698 | dronedarone |
|  |  |  | medication | NLM:RXNORM:19484 | bisoprolol |
|  |  |  | medication | NLM:RXNORM:20352 | carvedilol |
|  |  |  | medication | NLM:RXNORM:31555 | nebivolol |
|  |  |  | medication | NLM:RXNORM:49737 | esmolol |
|  |  |  | medication | NLM:RXNORM:6185 | labetalol |
|  |  |  | medication | NLM:RXNORM:6918 | metoprolol |
|  |  |  | medication | NLM:RXNORM:7226 | nadolol |
|  |  |  | medication | NLM:RXNORM:8332 | pindolol |
|  |  |  | medication | NLM:RXNORM:8787 | propranolol |
|  |  |  | medication | NLM:RXNORM:9947 | sotalol |
|  |  |  | medication | NLM:RXNORM:3443 | diltiazem |
|  |  |  | medication | NLM:RXNORM:11170 | verapamil |
|  |  |  | medication | NLM:RXNORM:3407 | digoxin |
| Group 3 | | | | | |
|  | **Group 3A Atrial Fibrillation** | | | | |
|  | must have | any of | diagnosis | UMLS:ICD10CM:I48.0 | Paroxysmal atrial fibrillation |
|  |  |  | diagnosis | UMLS:ICD10CM:I48.1 | Persistent atrial fibrillation |
|  |  |  | diagnosis | UMLS:ICD10CM:I48.2 | Chronic atrial fibrillation |
|  | date constraint | | This group occurred before 3 years ago | | |
|  | event relationship | | Any instance of medication occurred within 1 year on or after the first instance of Atrial Fibrillation | | |
|  | **Group 3B medication** | | | | |
|  | must have | any of | procedure | UMLS:CPT:93657 | Additional linear or focal intracardiac catheter ablation of the left or right atrium for treatment of atrial fibrillation remaining after completion of pulmonary vein isolation (List separately in addition to code for primary procedure) |
|  |  |  | procedure | UMLS:CPT:93656 | Comprehensive electrophysiologic evaluation including transseptal catheterizations, insertion and repositioning of multiple electrode catheters with intracardiac catheter ablation of atrial fibrillation by pulmonary vein isolation, including intracardiac electrophysiologic 3-dimensional mapping, intracardiac echocardiography including imaging supervision and interpretation, induction or attempted induction of an arrhythmia including left or right atrial pacing/recording, right ventricular pacing/recording, and His bundle recording, when performed |
| Group 4 | | | | | |
|  | **Group 4A Ablation** | | | | |
|  | must have | any of | diagnosis | UMLS:ICD10CM:I48.0 | Paroxysmal atrial fibrillation |
|  |  |  | diagnosis | UMLS:ICD10CM:I48.1 | Persistent atrial fibrillation |
|  |  |  | diagnosis | UMLS:ICD10CM:I48.2 | Chronic atrial fibrillation |
|  | date constraint | | This group occurred before 3 years ago | | |
|  | event relationship | | Any instance of Group 4B occurred within 1 year before or up to 1 year after any instance of Ablation | | |
|  | **Group 4B** | | | | |
|  | cannot have |  | diagnosis | UMLS:ICD10CM:F01-F09 | Mental disorders due to known physiological conditions |
|  |  | or | diagnosis | UMLS:ICD10CM:F20-F29 | Schizophrenia, schizotypal, delusional, and other non-mood psychotic disorders |
|  |  | or | diagnosis | UMLS:ICD10CM:F30-F39 | Mood [affective] disorders |
|  |  | or | diagnosis | UMLS:ICD10CM:F40-F48 | Anxiety, dissociative, stress-related, somatoform and other nonpsychotic mental disorders |

|  |
| --- |

### **Table S2:** Query Criteria for Cohort (query name: non-ablation group)

| Ungrouped terms | | | | | |
| --- | --- | --- | --- | --- | --- |
|  | must have |  | demographics | Age | Age (at least 18 years (most recent occurrence)) |
| Group 1 | | | | | |
|  | **visit** | | | | |
|  | must have |  | visit | TNX:Visit | Visit |
|  | number of occurrences | | Greater than or equal to 2 instances | | |
|  | date constraint | | The terms in this group occurred at any time | | |
| Group 2 | | | | | |
|  | **Group 2A Atrial Fibrillation** | | | | |
|  | must have | any of | diagnosis | UMLS:ICD10CM:I48.0 | Paroxysmal atrial fibrillation |
|  |  |  | diagnosis | UMLS:ICD10CM:I48.1 | Persistent atrial fibrillation |
|  |  |  | diagnosis | UMLS:ICD10CM:I48.2 | Chronic atrial fibrillation |
|  | date constraint | | This group occurred before 3 years ago | | |
|  | event relationship | | Any instance of medication occurred on or after the first instance of Atrial Fibrillation | | |
|  | **Group 2B medication** | | | | |
|  | must have | any of | medication | NLM:ATC:C01B | ANTIARRHYTHMICS, CLASS I AND III |
|  |  |  | medication | NLM:VA:CV300 | ANTIARRHYTHMICS |
|  |  |  | medication | NLM:ATC:C07 | BETA BLOCKING AGENTS |
|  |  |  | medication | NLM:VA:CV100 | BETA BLOCKERS/RELATED |
|  |  |  | medication | NLM:VA:CV100 | BETA BLOCKERS/RELATED |
|  |  |  | medication | NLM:VA:CV200 | CALCIUM CHANNEL BLOCKERS |
|  |  |  | medication | NLM:ATC:C08 | CALCIUM CHANNEL BLOCKERS |
|  |  |  | medication | NLM:ATC:C01A | CARDIAC GLYCOSIDES |
|  |  |  | medication | NLM:RXNORM:4441 | flecainide |
|  |  |  | medication | NLM:RXNORM:8754 | propafenone |
|  |  |  | medication | NLM:RXNORM:4441 | flecainide |
|  |  |  | medication | NLM:RXNORM:9947 | sotalol |
|  |  |  | medication | NLM:RXNORM:703 | amiodarone |
|  |  |  | medication | NLM:RXNORM:233698 | dronedarone |
|  |  |  | medication | NLM:RXNORM:19484 | bisoprolol |
|  |  |  | medication | NLM:RXNORM:20352 | carvedilol |
|  |  |  | medication | NLM:RXNORM:31555 | nebivolol |
|  |  |  | medication | NLM:RXNORM:49737 | esmolol |
|  |  |  | medication | NLM:RXNORM:6185 | labetalol |
|  |  |  | medication | NLM:RXNORM:6918 | metoprolol |
|  |  |  | medication | NLM:RXNORM:7226 | nadolol |
|  |  |  | medication | NLM:RXNORM:8332 | pindolol |
|  |  |  | medication | NLM:RXNORM:8787 | propranolol |
|  |  |  | medication | NLM:RXNORM:9947 | sotalol |
|  |  |  | medication | NLM:RXNORM:3443 | diltiazem |
|  |  |  | medication | NLM:RXNORM:11170 | verapamil |
|  |  |  | medication | NLM:RXNORM:3407 | digoxin |
| Group 3 | | | | | |
|  | **Group 3A Atrial Fibrillation** | | | | |
|  | must have | any of | diagnosis | UMLS:ICD10CM:I48.0 | Paroxysmal atrial fibrillation |
|  |  |  | diagnosis | UMLS:ICD10CM:I48.1 | Persistent atrial fibrillation |
|  |  |  | diagnosis | UMLS:ICD10CM:I48.2 | Chronic atrial fibrillation |
|  | date constraint | | This group occurred before 3 years ago | | |
|  | event relationship | | Any instance of medication occurred on or after the first instance of Atrial Fibrillation | | |
|  | **Group 3B medication** | | | | |
|  | cannot have |  | procedure | UMLS:CPT:93657 | Additional linear or focal intracardiac catheter ablation of the left or right atrium for treatment of atrial fibrillation remaining after completion of pulmonary vein isolation (List separately in addition to code for primary procedure) |
|  |  | or | procedure | UMLS:CPT:93656 | Comprehensive electrophysiologic evaluation including transseptal catheterizations, insertion and repositioning of multiple electrode catheters with intracardiac catheter ablation of atrial fibrillation by pulmonary vein isolation, including intracardiac electrophysiologic 3-dimensional mapping, intracardiac echocardiography including imaging supervision and interpretation, induction or attempted induction of an arrhythmia including left or right atrial pacing/recording, right ventricular pacing/recording, and His bundle recording, when performed |
| Group 4 | | | | | |
|  | **Group 4A Ablation** | | | | |
|  | must have | any of | diagnosis | UMLS:ICD10CM:I48.0 | Paroxysmal atrial fibrillation |
|  |  |  | diagnosis | UMLS:ICD10CM:I48.1 | Persistent atrial fibrillation |
|  |  |  | diagnosis | UMLS:ICD10CM:I48.2 | Chronic atrial fibrillation |
|  | date constraint | | This group occurred before 3 years ago | | |
|  | event relationship | | Any instance of Group 4B occurred within 1 year before or up to 1 year after any instance of Ablation | | |
|  | **Group 4B** | | | | |
|  | cannot have |  | diagnosis | UMLS:ICD10CM:F01-F09 | Mental disorders due to known physiological conditions |
|  |  | or | diagnosis | UMLS:ICD10CM:F20-F29 | Schizophrenia, schizotypal, delusional, and other non-mood psychotic disorders |
|  |  | or | diagnosis | UMLS:ICD10CM:F30-F39 | Mood [affective] disorders |
|  |  | or | diagnosis | UMLS:ICD10CM:F40-F48 | Anxiety, dissociative, stress-related, somatoform and other nonpsychotic mental disorders |

## Analysis Setup

This section contains the Index Event and Time Window definitions and a list of selected outcomes and the analyses.

### Index Event & Time Window Definitions

The index event defines the point in time when each patient in the cohort enters the analysis. To define an index event for the cohort, one or more criteria for the cohort must be selected. The index date for each patient within a cohort is the day on which the patient first met the selected criteria for the cohort (listed in the table below).

As the index event defines the earliest time point after which outcomes are analyzed, the time window defines the duration during which outcomes are analyzed. The time window can start on the same day as the index event or at any specified time interval after the index event. The time window can end any time after the start date. Outcomes are defined as diagnoses, medications, procedures, or laboratory values that happened in the time window starting after the first occurrence of the index event.

### Time Window Used in this Analysis

This analysis included outcomes that occurred in the time window that started 90 days after the first occurrence of the index event and ended 180 days after the first occurrence of the index event

The index event only includes events that occurred up to 20 years ago. Patients whose index event occurred 20 years or more ago are excluded. In this analysis, 0 patients in Cohort 1 and 0 patients in Cohort 2 were excluded because they met the index event more than 20 years ago.

### Index Events Used in this Analysis

Index events for the Compare Outcomes analysis were derived from the cohort definitions. Index events were defined separately for each cohort and were based on the criteria used in the original cohort definition.

The index event for Cohort was defined as the following:

| Table S3. The index event for Cohort 1 (query name: ablation group) was defined as the following:   \|  \| \| \| \| \| \| \| --- \| --- \| --- \| --- \| --- \| --- \| \| Group 1 \| \| \| \| \| \| \|  \| **Group 1A Atrial Fibrillation** \| \| \| \| \| \|  \| must have \| any of \| diagnosis \| UMLS:ICD10CM:I48.0 \| Paroxysmal atrial fibrillation \| \|  \|  \|  \| diagnosis \| UMLS:ICD10CM:I48.1 \| Persistent atrial fibrillation \| \|  \|  \|  \| diagnosis \| UMLS:ICD10CM:I48.2 \| Chronic atrial fibrillation \| \|  \| date constraint \| \| This group occurred before 3 years ago \| \| \| \|  \| event relationship \| \| Any instance of medication occurred within 1 year on or after the first instance of Atrial Fibrillation \| \| \| \|  \| **Group 1B medication** \| \| \| \| \| \|  \| must have \| any of \| procedure \| UMLS:CPT:93657 \| Additional linear or focal intracardiac catheter ablation of the left or right atrium for treatment of atrial fibrillation remaining after completion of pulmonary vein isolation (List separately in addition to code for primary procedure) \| \|  \|  \|  \| procedure \| UMLS:CPT:93656 \| Comprehensive electrophysiologic evaluation including transseptal catheterizations, insertion and repositioning of multiple electrode catheters with intracardiac catheter ablation of atrial fibrillation by pulmonary vein isolation, including intracardiac electrophysiologic 3-dimensional mapping, intracardiac echocardiography including imaging supervision and interpretation, induction or attempted induction of an arrhythmia including left or right atrial pacing/recording, right ventricular pacing/recording, and His bundle recording, when performed \| | | | | | |
| --- | --- | --- | --- | --- | --- | --- | --- | --- | --- | --- | --- | --- | --- | --- | --- | --- | --- | --- | --- | --- | --- | --- | --- | --- | --- | --- | --- | --- | --- | --- | --- | --- | --- | --- | --- | --- | --- | --- | --- | --- | --- | --- | --- | --- | --- | --- | --- | --- | --- | --- | --- | --- | --- | --- | --- | --- | --- | --- | --- | --- | --- | --- | --- | --- | --- | --- | --- | --- | --- | --- | --- |
| Table S4. The index event for Cohort 2 (query name: non-ablation group) was defined as the following: | | | | | |
|  | | | | | |
| Group 1 | | | | | |
|  | **Group 1A Atrial Fibrillation** | | | | |
|  | must have | any of | diagnosis | UMLS:ICD10CM:I48.0 | Paroxysmal atrial fibrillation |
|  |  |  | diagnosis | UMLS:ICD10CM:I48.1 | Persistent atrial fibrillation |
|  |  |  | diagnosis | UMLS:ICD10CM:I48.2 | Chronic atrial fibrillation |
|  | date constraint | | This group occurred before 3 years ago | | |
|  | event relationship | | Any instance of medication occurred on or after the first instance of Atrial Fibrillation | | |
|  | **Group 1B medication** | | | | |
|  | cannot have |  | procedure | UMLS:CPT:93657 | Additional linear or focal intracardiac catheter ablation of the left or right atrium for treatment of atrial fibrillation remaining after completion of pulmonary vein isolation (List separately in addition to code for primary procedure) |
|  |  | or | procedure | UMLS:CPT:93656 | Comprehensive electrophysiologic evaluation including transseptal catheterizations, insertion and repositioning of multiple electrode catheters with intracardiac catheter ablation of atrial fibrillation by pulmonary vein isolation, including intracardiac electrophysiologic 3-dimensional mapping, intracardiac echocardiography including imaging supervision and interpretation, induction or attempted induction of an arrhythmia including left or right atrial pacing/recording, right ventricular pacing/recording, and His bundle recording, when performed |

### Survival Analysis

The Kaplan-Meier Analysis estimates probability of the outcome at a respective time interval (daily time interval is used in this analysis). In order to account for the patients who exited the cohort during the analysis period, and therefore should not be included in the analysis, censoring is applied. In this analysis, patients are removed from the analysis (censored) after the last fact in their record.

### **Table S5:** Outcome Definitions

Table below outlines the definitions for each outcome and the analysis specifications. For outcome definitions consisting of more than one term, at least one term must match.

| Any psychiatric disorders | | | | |
| --- | --- | --- | --- | --- |
|  | **Outcome definition** | | | |
|  | | Diagnosis | UMLS:ICD10CM:F31.4 | Bipolar disorder, current episode depressed, severe, without psychotic features |
|  | | Diagnosis | UMLS:ICD10CM:F31.5 | Bipolar disorder, current episode depressed, severe, with psychotic features |
|  | | Diagnosis | UMLS:ICD10CM:F31.30 | Bipolar disorder, current episode depressed, mild or moderate severity, unspecified |
|  | | Diagnosis | UMLS:ICD10CM:F31.31 | Bipolar disorder, current episode depressed, mild |
|  | | Diagnosis | UMLS:ICD10CM:F31.32 | Bipolar disorder, current episode depressed, moderate |
|  | | Diagnosis | UMLS:ICD10CM:F43.21 | Adjustment disorder with depressed mood |
|  | | Diagnosis | UMLS:ICD10CM:F33.0 | Major depressive disorder, recurrent, mild |
|  | | Diagnosis | UMLS:ICD10CM:F33.1 | Major depressive disorder, recurrent, moderate |
|  | | Diagnosis | UMLS:ICD10CM:F33.2 | Major depressive disorder, recurrent severe without psychotic features |
|  | | Diagnosis | UMLS:ICD10CM:F33.3 | Major depressive disorder, recurrent, severe with psychotic symptoms |
|  | | Diagnosis | UMLS:ICD10CM:F33.9 | Major depressive disorder, recurrent, unspecified |
|  | | Diagnosis | UMLS:ICD10CM:F41 | Other anxiety disorders |
|  | | Diagnosis | UMLS:ICD10CM:G47.0 | Insomnia |
|  | **Settings for the performed analyses** | | | |
|  | | Kaplan - Meier survival analysis | | including patients with outcome prior to the time window |
| anxiety | | | | |
|  | **Outcome definition** | | | |
|  | | Diagnosis | UMLS:ICD10CM:F41 | Other anxiety disorders |
|  | | Diagnosis | UMLS:ICD10CM:F43.22 | Adjustment disorder with anxiety |
|  | | Diagnosis | UMLS:ICD10CM:F43.23 | Adjustment disorder with mixed anxiety and depressed mood |
|  | **Settings for the performed analyses** | | | |
|  | | Kaplan - Meier survival analysis | | including patients with outcome prior to the time window |
| depression | | | | |
|  | **Outcome definition** | | | |
|  | | Diagnosis | UMLS:ICD10CM:F31.4 | Bipolar disorder, current episode depressed, severe, without psychotic features |
|  | | Diagnosis | UMLS:ICD10CM:F31.5 | Bipolar disorder, current episode depressed, severe, with psychotic features |
|  | | Diagnosis | UMLS:ICD10CM:F32.0 | Major depressive disorder, single episode, mild |
|  | | Diagnosis | UMLS:ICD10CM:F32.1 | Major depressive disorder, single episode, moderate |
|  | | Diagnosis | UMLS:ICD10CM:F32.2 | Major depressive disorder, single episode, severe without psychotic features |
|  | | Diagnosis | UMLS:ICD10CM:F32.3 | Major depressive disorder, single episode, severe with psychotic features |
|  | | Diagnosis | UMLS:ICD10CM:F32.9 | Major depressive disorder, single episode, unspecified |
|  | | Diagnosis | UMLS:ICD10CM:F32.A | Depression, unspecified |
|  | | Diagnosis | UMLS:ICD10CM:F33.0 | Major depressive disorder, recurrent, mild |
|  | | Diagnosis | UMLS:ICD10CM:F33.1 | Major depressive disorder, recurrent, moderate |
|  | | Diagnosis | UMLS:ICD10CM:F33.2 | Major depressive disorder, recurrent severe without psychotic features |
|  | | Diagnosis | UMLS:ICD10CM:F33.3 | Major depressive disorder, recurrent, severe with psychotic symptoms |
|  | | Diagnosis | UMLS:ICD10CM:F33.8 | Other recurrent depressive disorders |
|  | | Diagnosis | UMLS:ICD10CM:F33.9 | Major depressive disorder, recurrent, unspecified |
|  | | Diagnosis | UMLS:ICD10CM:F34.1 | Dysthymic disorder |
|  | | Diagnosis | UMLS:ICD10CM:F31.30 | Bipolar disorder, current episode depressed, mild or moderate severity, unspecified |
|  | | Diagnosis | UMLS:ICD10CM:F31.31 | Bipolar disorder, current episode depressed, mild |
|  | | Diagnosis | UMLS:ICD10CM:F31.32 | Bipolar disorder, current episode depressed, moderate |
|  | **Settings for the performed analyses** | | | |
|  | | Kaplan - Meier survival analysis | | including patients with outcome prior to the time window |
| insomina | | | | |
|  | **Outcome definition** | | | |
|  | | Diagnosis | UMLS:ICD10CM:G47.0 | Insomnia |
|  | | Diagnosis | UMLS:ICD10CM:G47.00 | Insomnia, unspecified |
|  | | Diagnosis | UMLS:ICD10CM:G47.01 | Insomnia due to medical condition |
|  | | Diagnosis | UMLS:ICD10CM:F51.01 | Primary insomnia |
|  | **Settings for the performed analyses** | | | |
|  | | Kaplan - Meier survival analysis | | including patients with outcome prior to the time window |
| negative control | | | | |
|  | **Outcome definition** | | | |
|  | | Diagnosis | UMLS:ICD10CM:L20 | Atopic dermatitis |
|  | **Settings for the performed analyses** | | | |
|  | | Kaplan - Meier survival analysis | | including patients with outcome prior to the time window |
| positive control | | | | |
|  | **Outcome definition** | | | |
|  | | Diagnosis | UMLS:ICD10CM:I63 | Cerebral infarction |
|  | **Settings for the performed analyses** | | | |
|  | | Kaplan - Meier survival analysis | | including patients with outcome prior to the time window |
| Suicide ideations or attempts | | | | |
|  | **Outcome definition** | | | |
|  | | Diagnosis | UMLS:ICD10CM:T14.91 | Suicide attempt |
|  | | Diagnosis | UMLS:ICD10CM:T14.91XA | Suicide attempt, initial encounter |
|  | | Diagnosis | UMLS:ICD10CM:T14.91XD | Suicide attempt, subsequent encounter |
|  | | Diagnosis | UMLS:ICD10CM:T14.91XS | Suicide attempt, sequela |
|  | | Diagnosis | UMLS:ICD10CM:R45.851 | Suicidal ideations |
|  | | Diagnosis | UMLS:ICD10CM:F48 | Other nonpsychotic mental disorders |
|  | **Settings for the performed analyses** | | | |
|  | | Kaplan - Meier survival analysis | | including patients with outcome prior to the time window |
| dementia | | | | |
|  | **Outcome definition** | | | |
|  | | Diagnosis | UMLS:ICD10CM:F01 | Vascular dementia |
|  | | Diagnosis | UMLS:ICD10CM:F02 | Dementia in other diseases classified elsewhere |
|  | | Diagnosis | UMLS:ICD10CM:F03 | Unspecified dementia |
|  | | Diagnosis | UMLS:ICD10CM:G30 | Alzheimer's disease |
|  | | Medication | NLM:RXNORM:135447 | donepezil |
|  | | Medication | NLM:RXNORM:6719 | memantine |
|  | | Medication | NLM:RXNORM:183379 | rivastigmine |
|  | | Medication | NLM:RXNORM:4637 | galantamine |
|  | **Settings for the performed analyses** | | | |
|  | | Kaplan - Meier survival analysis | | including patients with outcome prior to the time window |

**Supplementary figures**

Figure S1. Subgroup analyses of the outcome of anxiety.
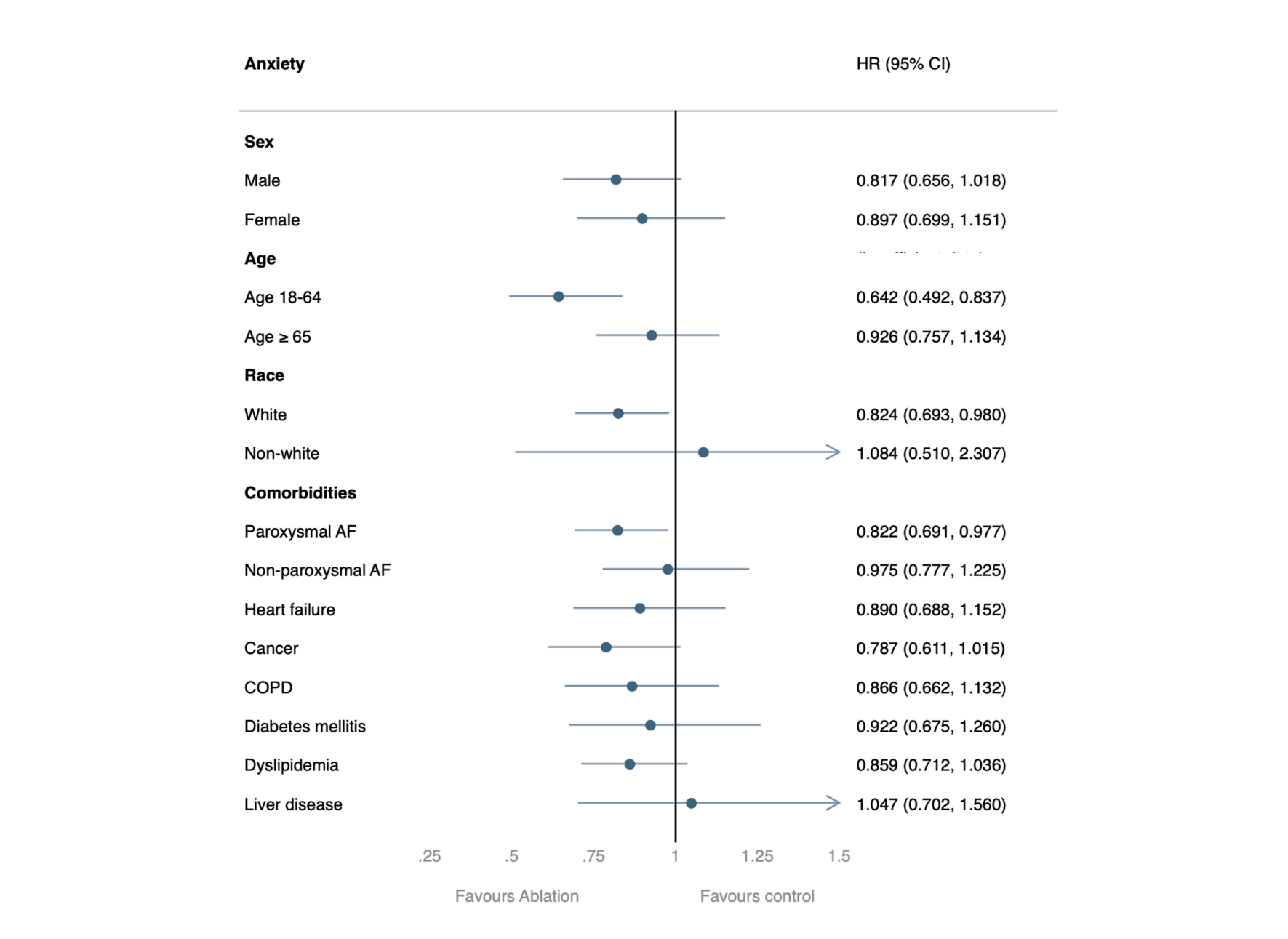


Figure S2. Subgroup analyses of the outcome of depression


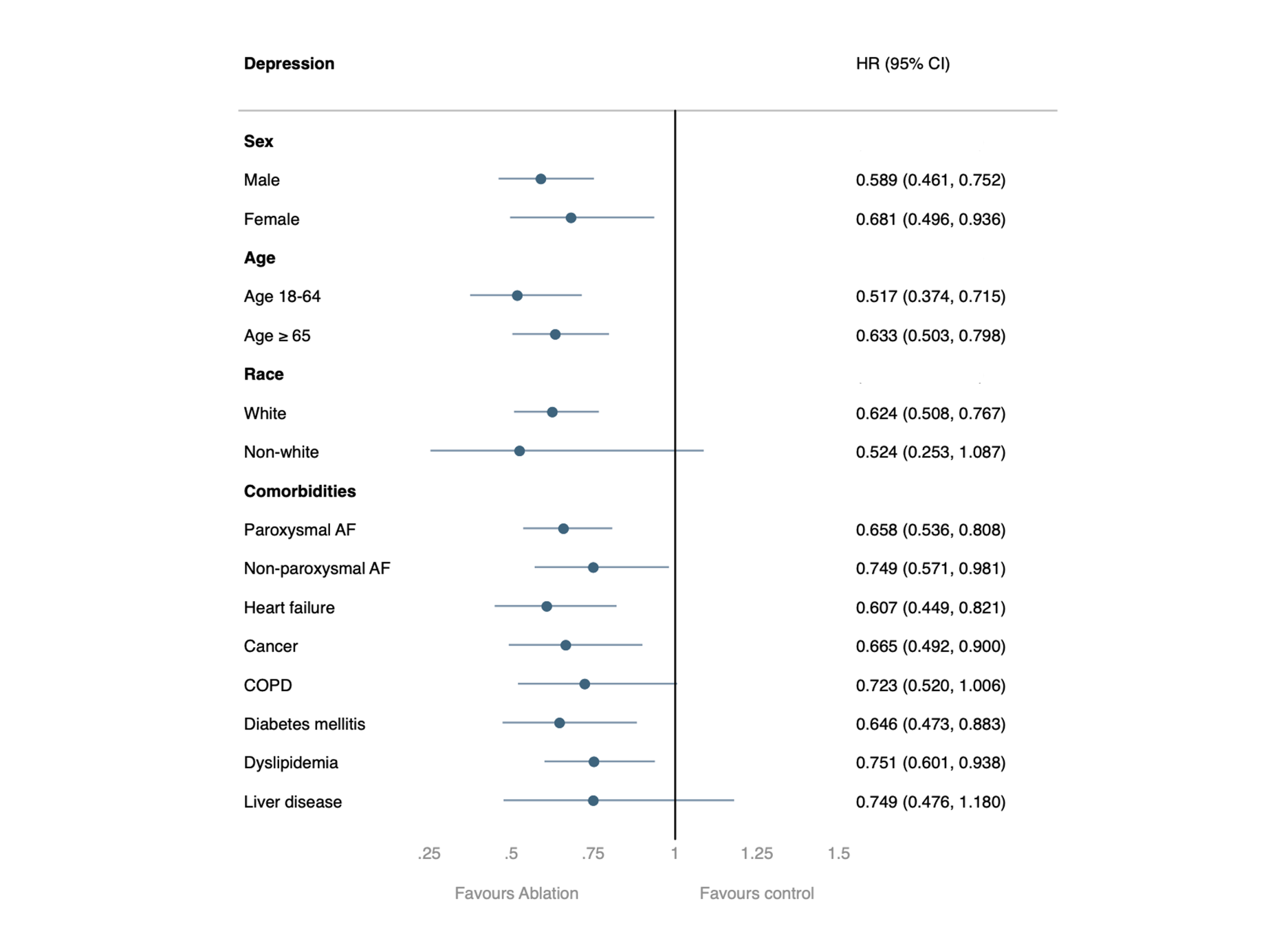


Figure S3. Subgroup analyses of the outcome of insomnia


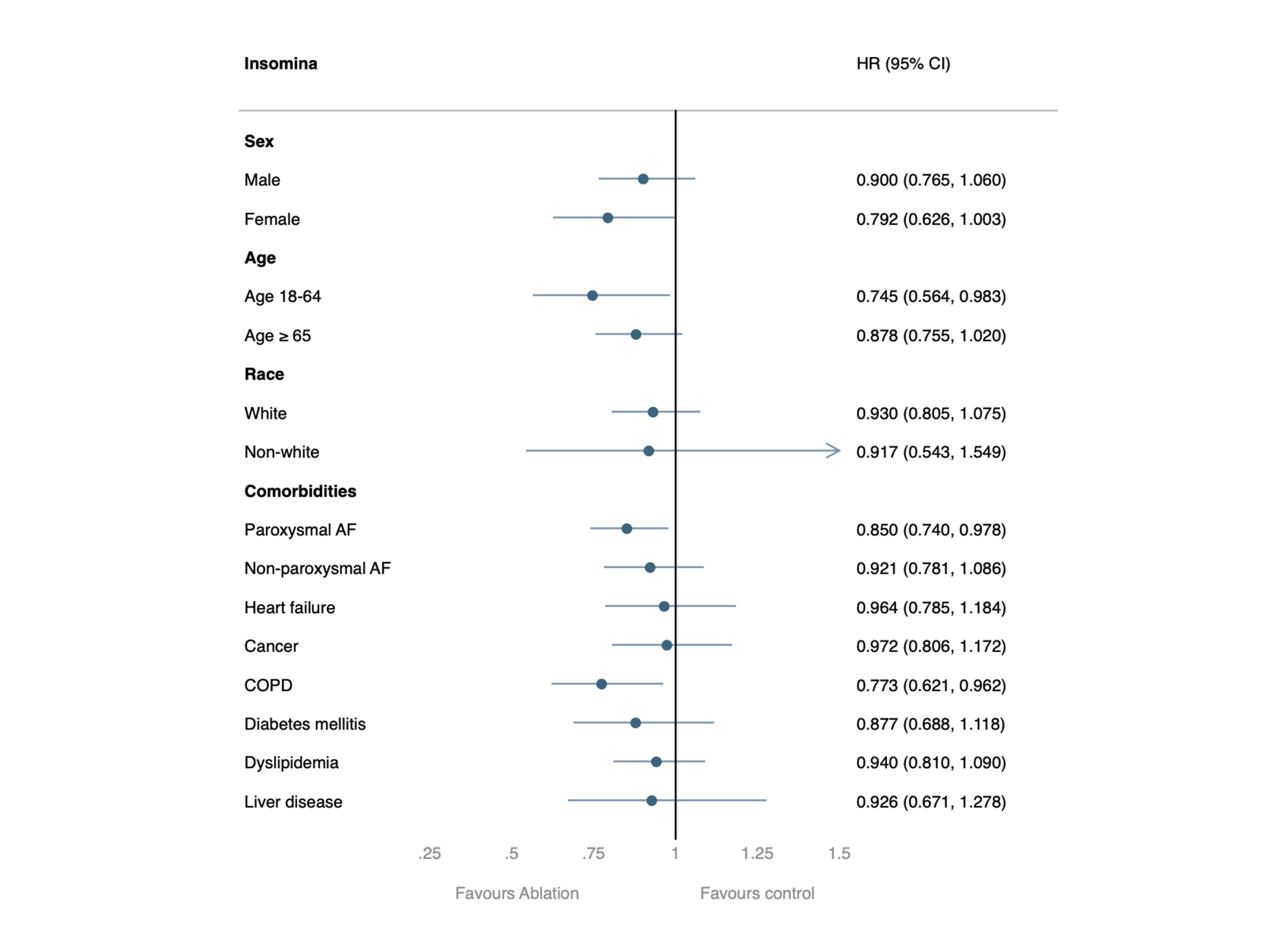


Figure S4. Subgroup analyses of the outcome of suicide ideation or attempt


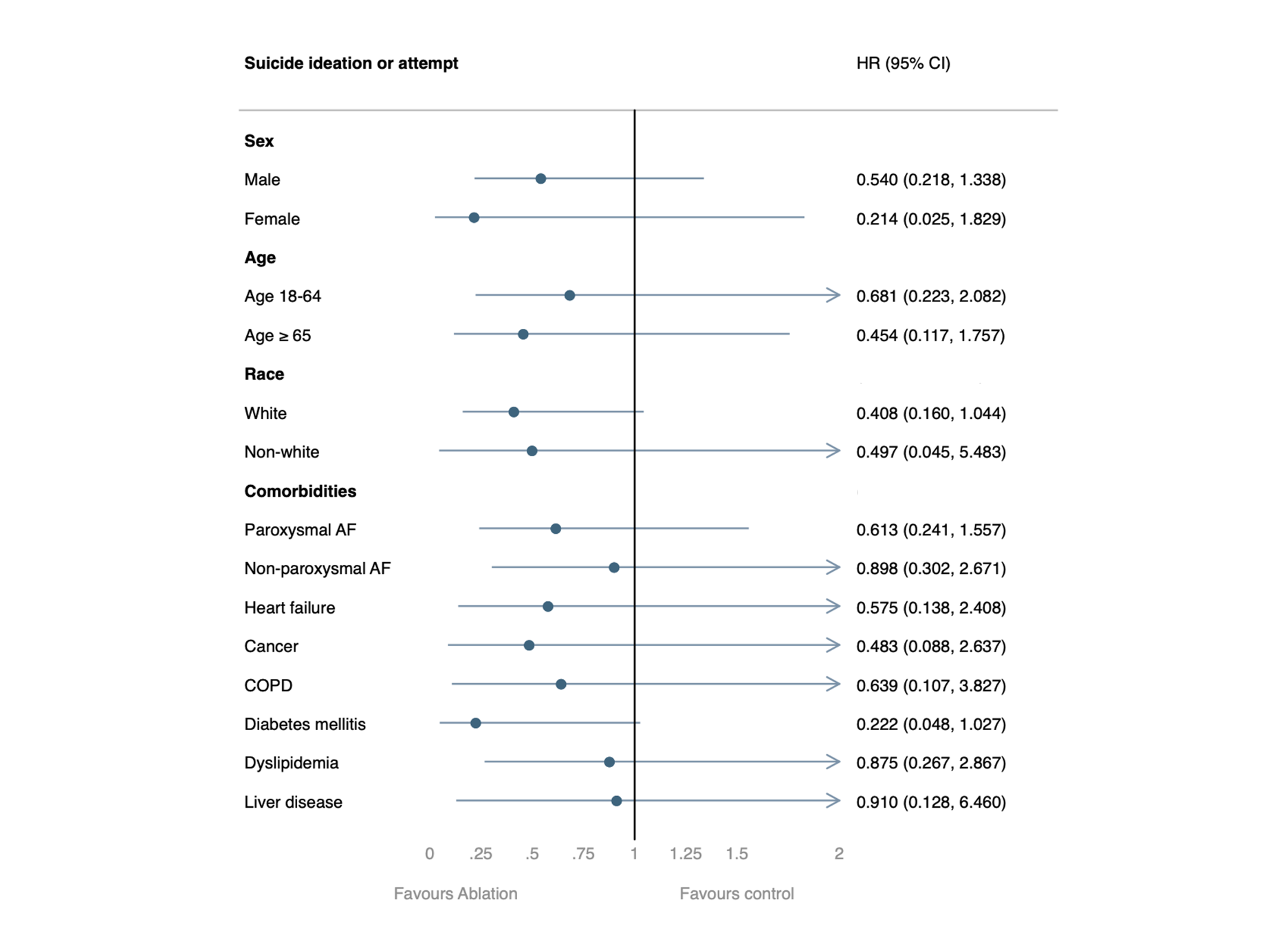


Figure S5. Subgroup analyses of the outcome of dementia
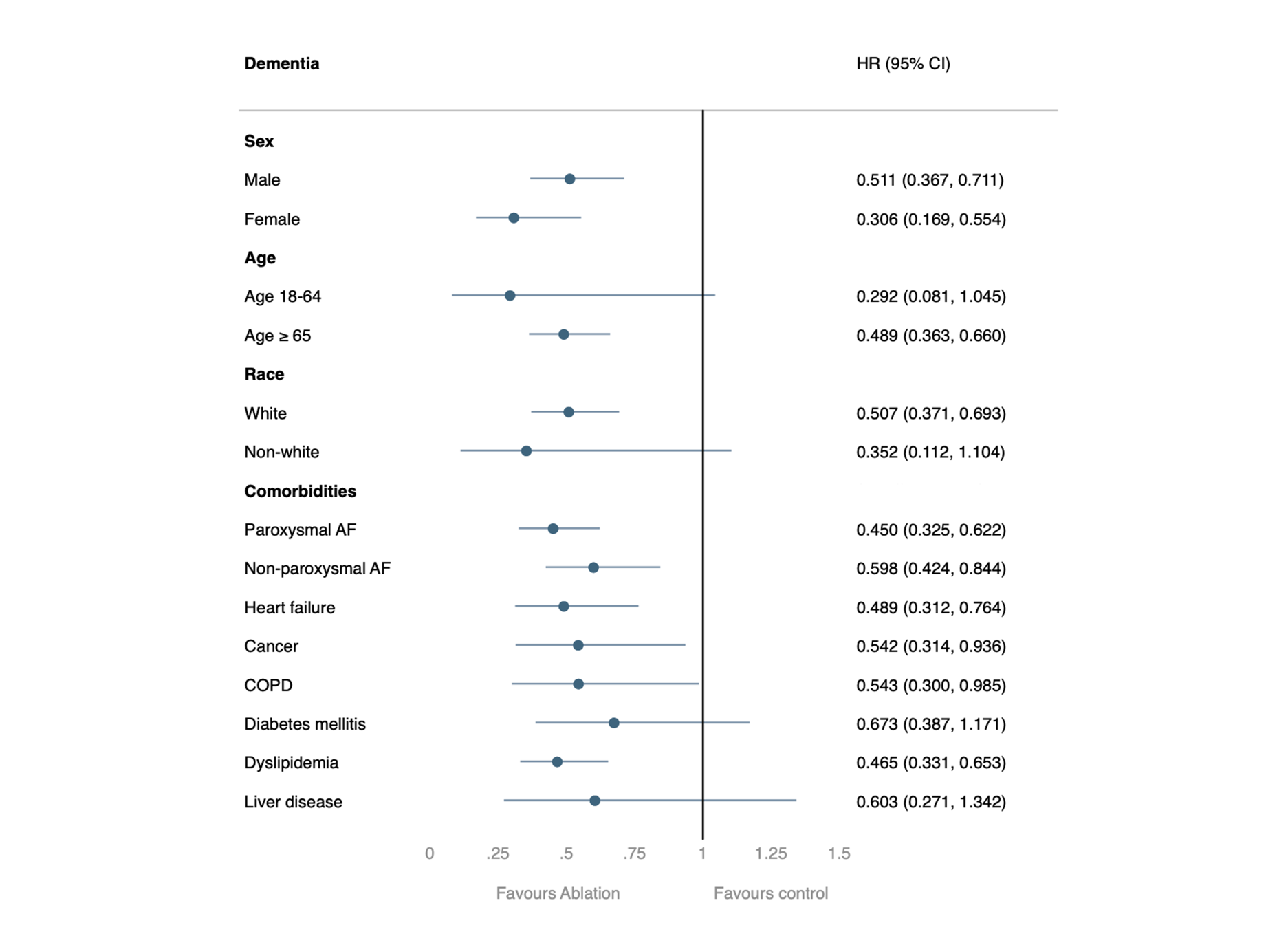


Figure S6. Subgroup analyses of the outcome of atopic dermatitis
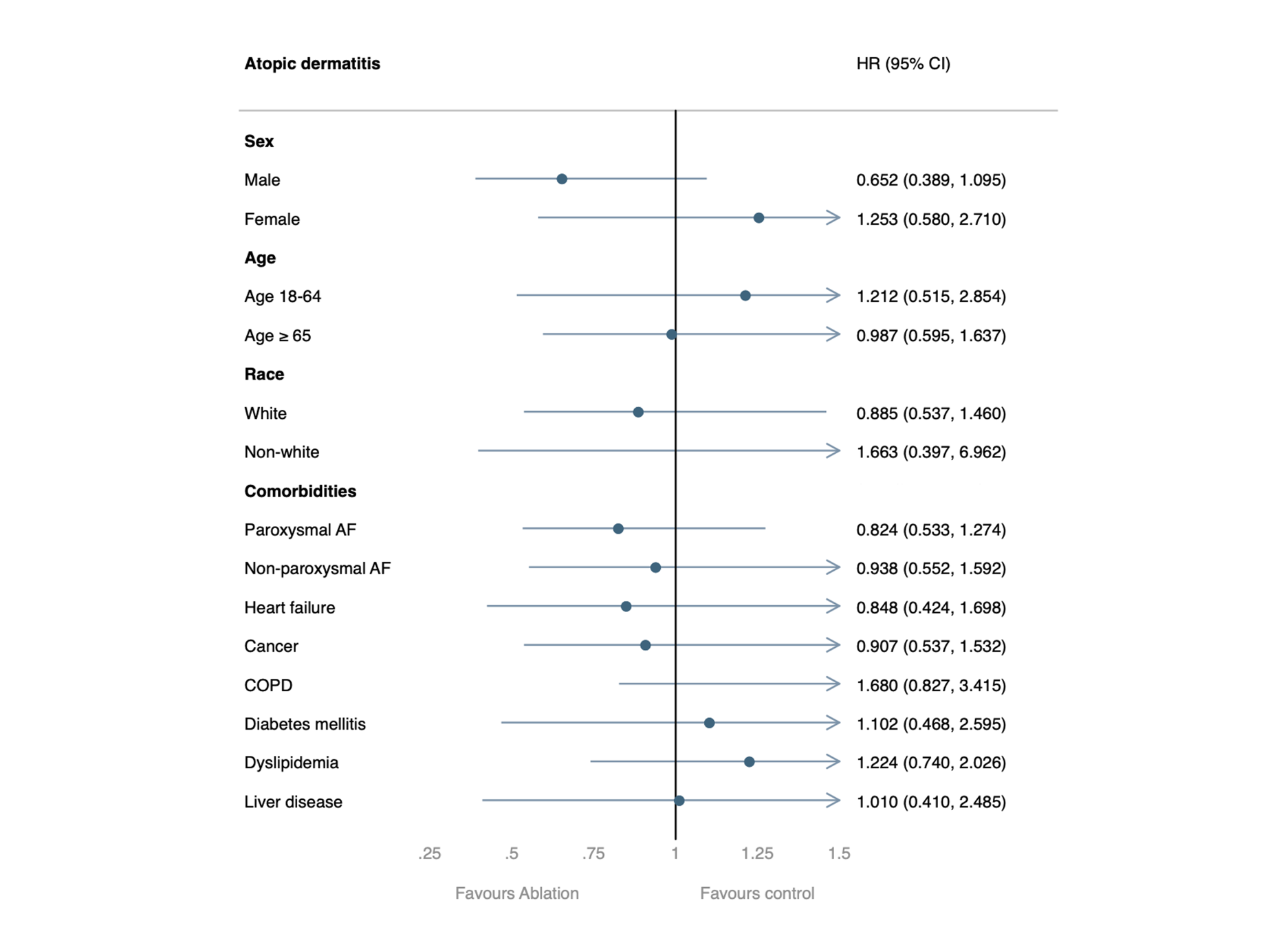


Figure S7. Subgroup analyses of the outcome of cerebral infarction
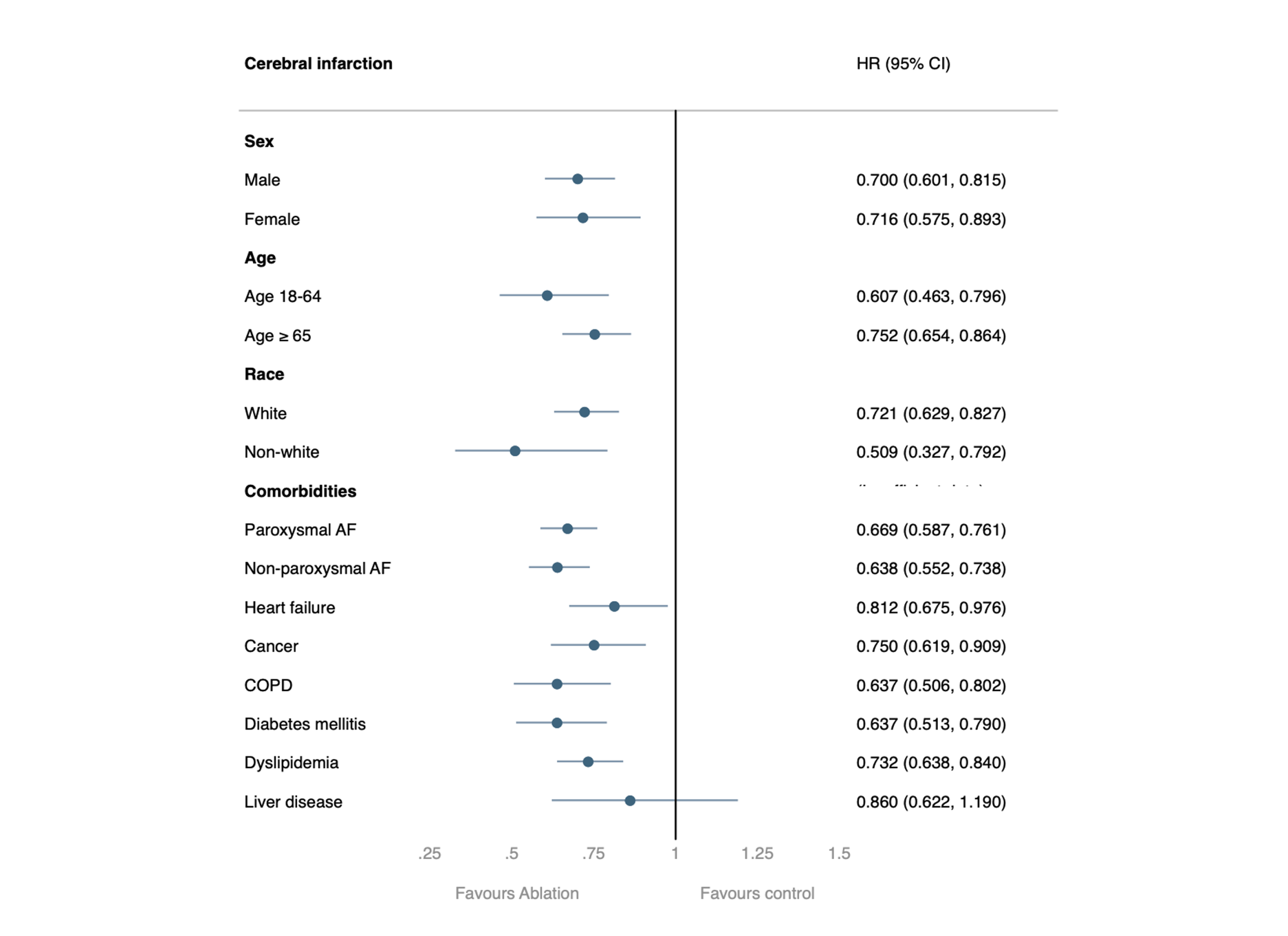

Supplement: Supplementary file 1 [file SupplementaryFile1.docx]
